# A *KCNK16* mutation causing TALK-1 gain of function is associated with maturity-onset diabetes of the young

**DOI:** 10.1172/jci.insight.138057

**Published:** 2021-07-08

**Authors:** Sarah M. Graff, Stephanie R. Johnson, Paul J. Leo, Prasanna K. Dadi, Matthew T. Dickerson, Arya Y. Nakhe, Aideen M. McInerney-Leo, Mhairi Marshall, Karolina E. Zaborska, Charles M. Schaub, Matthew A. Brown, David A. Jacobson, Emma L. Duncan

**Affiliations:** 1 Department of Molecular Physiology and Biophysics, Vanderbilt University, Nashville, Tennessee, USA.; 2 Department of Endocrinology, Queensland Children’s Hospital, South Brisbane, Queensland, Australia.; 3 Translational Genomics Group, Institute of Health and Biomedical Innovation, Faculty of Health, Queensland University of Technology, Translational Research Institute, Princess Alexandra Hospital, Woolloongabba, Queensland, Australia.; 4 Faculty of Medicine, University of Queensland, Herston, Queensland, Australia.; 5 Dermatology Research Centre, Dermatology Research Centre, The University of Queensland Diamantina Institute, Brisbane, Queensland, Australia.; 6 Guy’s and St Thomas’ NHS Foundation Trust and King’s College London NIHR Biomedical Research Centre, King’s College London, London, United Kingdom.; 7 Department of Twin Research & Genetic Epidemiology, School of Life Course Sciences, Faculty of Life Sciences and Medicine, King’s College London, London, United Kingdom.

**Keywords:** Endocrinology, Genetics, Calcium signaling, Diabetes, Ion channels

## Abstract

Maturity-onset diabetes of the young (MODY) is a heterogeneous group of monogenic disorders of impaired pancreatic β cell function. The mechanisms underlying MODY include β cell KATP channel dysfunction (e.g., *KCNJ11* [MODY13] or *ABCC8* [MODY12] mutations); however, no other β cell channelopathies have been associated with MODY to date. Here, we have identified a nonsynonymous coding variant in *KCNK16* (NM_001135105: c.341T>C, p.Leu114Pro) segregating with MODY. KCNK16 is the most abundant and β cell–restricted K^+^ channel transcript, encoding the two-pore-domain K^+^ channel TALK-1. Whole-cell K^+^ currents demonstrated a large gain of function with TALK-1 Leu114Pro compared with TALK-1 WT, due to greater single-channel activity. Glucose-stimulated membrane potential depolarization and Ca^2+^ influx were inhibited in mouse islets expressing TALK-1 Leu114Pro with less endoplasmic reticulum Ca^2+^ storage. TALK-1 Leu114Pro significantly blunted glucose-stimulated insulin secretion compared with TALK-1 WT in mouse and human islets. These data suggest that *KCNK16* is a previously unreported gene for MODY.

## Introduction

Maturity-onset diabetes of the young (MODY) is a rare monogenic cause of familial diabetes. To date, 11 genes have been confirmed to cause MODY, all of which are involved in pancreatic β cell insulin secretion and all with autosomal dominant transmission (1, 2). 2%–2.5% of pediatric diabetes cases carry pathogenic/likely pathogenic variants in MODY genes (3, 4); however, MODY is often undiagnosed, either because the diagnosis is not considered (5) or because genetic screening is limited. There are also cases with compelling clinical histories, in whom, despite comprehensive screening of known MODY genes, a genetic diagnosis cannot be made (4), suggesting as-yet-unidentified genetic cause(s).

β Cell glucose-stimulated insulin secretion (GSIS) is dependent on Ca^2+^ influx through voltage-dependent calcium channels (VDCC) (6, 7). Reduced Ca^2+^ influx decreases GSIS; thus, mutations that disrupt β cell Ca^2+^ entry can cause MODY or the closely related condition, neonatal diabetes (8, 9). For example, gain-of-function mutations in K_ATP_ channel subunits hyperpolarize the β cell membrane potential (*V_m_*), reducing VDCC activity, Ca^2+^ influx, and GSIS (8, 9). Other β cell K^+^ channels, including 2-pore domain K^+^ channels (K2P), also affect VDCC activity (10). Expression of *KCNK16*, which encodes TWIK-related alkaline pH–activated K2P (TALK-1) (11), is the most abundant and β cell–selective of all human K^+^ channel transcripts (12, 13), and TALK-1 gain-of-function mutations would be predicted to cause diabetes similarly (10).

Here, we have used exome sequencing to identify the first family of whom we are aware with MODY due to a mutation in *KCNK16*. The Leu114Pro substitution in TALK-1 affects the K^+^ selectivity filter, causing a profound increase in K^+^ current, altering β cell Ca^2+^ flux, and decreasing GSIS in both human and mouse islet cells.

## Results

### Pedigree.

**Figure 1:**
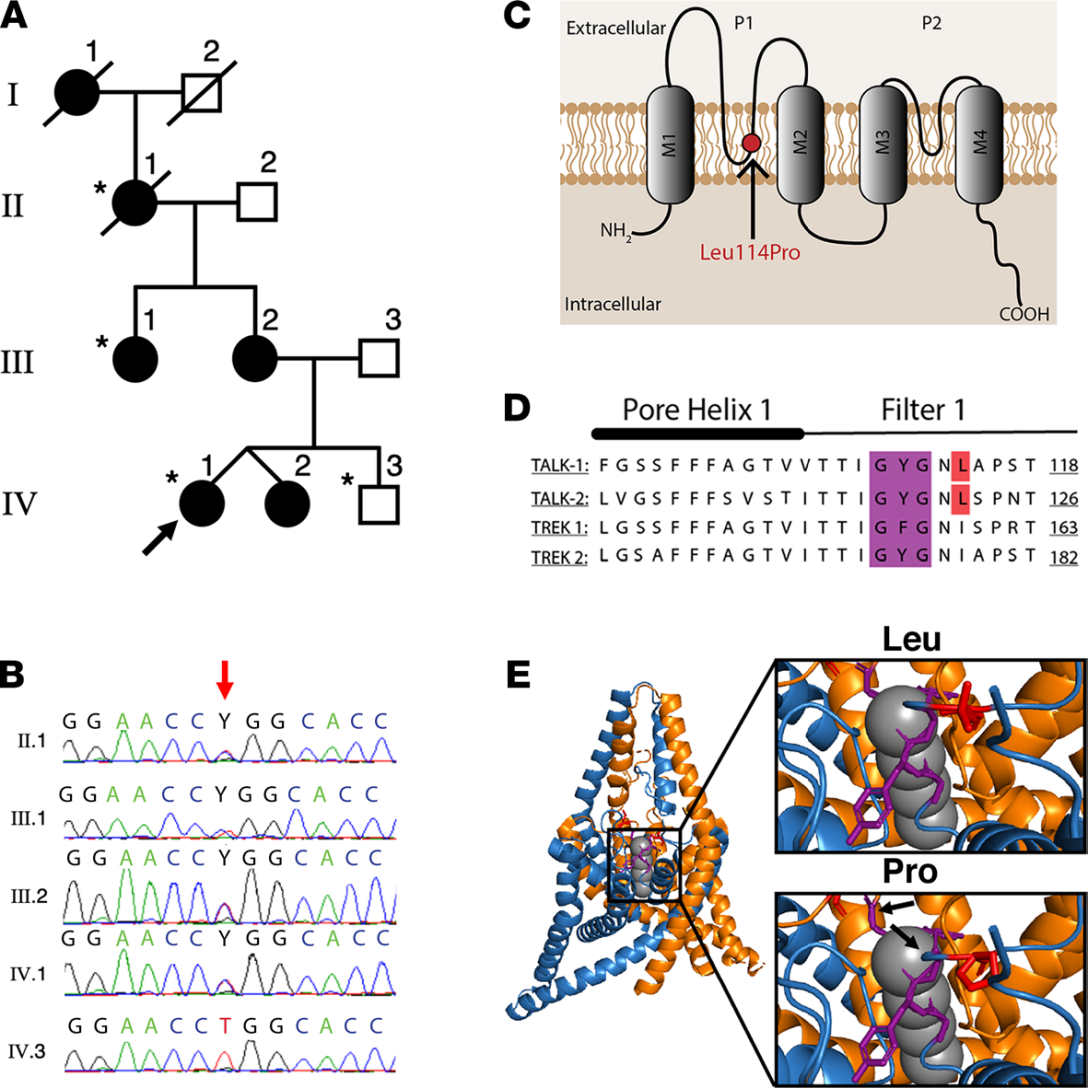
A *KCNK16* mutation cosegregates with MODY in a 4-generation family and is predicted to affect the K^+^ selectivity channel of TALK-1. (**A**) Family pedigree (asterisks indicate individuals undergoing exome sequencing; black shapes indicate individuals with diabetes; arrow indicates proband), (**B**) with chromatogram of *KCNK16* variant (c.341T>C) (red arrow indicates variant). (**C**) Location of the predicted protein change (p.Leu114Pro), within the first pore domain and K^+^ selectivity channel of TALK-1, (**D**) with alignment of pore helix 1 and filter 1 amino acid sequences of *KCNK16* with other KCNK channels (mutation position indicated in red; selectivity filter indicated in purple). (**E**) Predicted conformational shifts (indicated by the arrows) in the K^+^ selectivity filter, modeled using TREK2 crystalline structure.

A 4-generation family, with 6 affected family members and apparent autosomal dominant diabetes (Figure 1A), was identified through a nonobese proband, who initially presented, aged 15 years, with elevated fasting plasma glucose (7.8 mmol/L) and an abnormal oral glucose tolerance test (glucose 19.6 mmol/L 2 hours after 75 g glucose). Antibody testing (islet cell, islet antigen 2, and glutamic acid decarboxylase-65) was negative. Sanger sequencing for mutations in *GCK*, *HNF1A*, and *HNF4A* (the most common MODY genes) was negative. Over two decades, the proband required minimal insulin to maintain HbA1c of 5.7%–6.5%, and she did not experience ketosis or other diabetes-related complications. She successfully transitioned to diet and metformin; however, during pregnancy she required insulin. Her twin sister, mother, maternal aunt, maternal grandmother also had insulin-requiring diabetes (from 8–12 units to 20–25 units daily). None had experienced ketosis or other diabetes-related complications (detailed in Supplemental Results *Extended Clinical Data*; supplemental material available online with this article; https://doi.org/10.1172/jci.insight.138057DS1). Her maternal great-grandmother also had diabetes.

### Exome sequencing in a family with MODY identifies a previously unreported coding variant in KCNK16.

Initial exome sequencing and analysis of known MODY genes in the proband identified a splice site mutation in *ABCC8* (NM_000352 c.1332+4 delC). However, this variant was not predicted to affect splicing (14) and, when assessed by Sanger sequencing, did not segregate appropriately in the pedigree. Subsequent exome sequencing and analysis of the extended pedigree identified previously unreported good-quality coding variants in 2 genes, *KCNK16* and *USP42*, with appropriate segregation (Figure 1, A and B; Supplemental Table 2; and Supplemental Table 3). Ubiquitin-specific peptidase 42 (*USP42*) is involved in spermatogenesis (15) and is not expressed in the pancreas (16); it was considered an unlikely MODY candidate. However, potassium channel, subfamily K, member 16 (*KCNK16*) encodes TALK-1, which has an established role in GSIS (10). Further, the *KCNK16*-containing locus is associated with T2DM (10, 17, 18).

The *KCNK16* variant (NM_001135105: c.341T>C) has not previously been reported in gnomAD (https://gnomad.broadinstitute.org/, accessed January, 22 2021), 1000Genomes (http://www.1000genomes.org), or dbSNP137 (http://www.ncbi.nlm.nih.gov/projects/SNP/). It affects a highly conserved base (genome evolutionary rate profiling score of 5.65), with the resultant amino acid change (p.Leu114Pro) predicted to involve the pore domain 1 of TALK-1, immediately downstream of the GYG K^+^ selectivity filter (Figure 1C). The GYG motif, and leucine 114 specifically, shows strong sequence homology with other K2P channels (Figure 1D). As the crystal structure of TALK-1 is unpublished, TREK-2 was used to model the p.Leu114Pro mutation, which demonstrated a conformational shift in both the GYG motif and pore domain (Figure 1E), strongly suggesting that TALK-1 Leu114Pro would significantly affect K^+^ permeability.

### TALK-1 Leu114Pro results in a gain of function.

**Figure 2:**
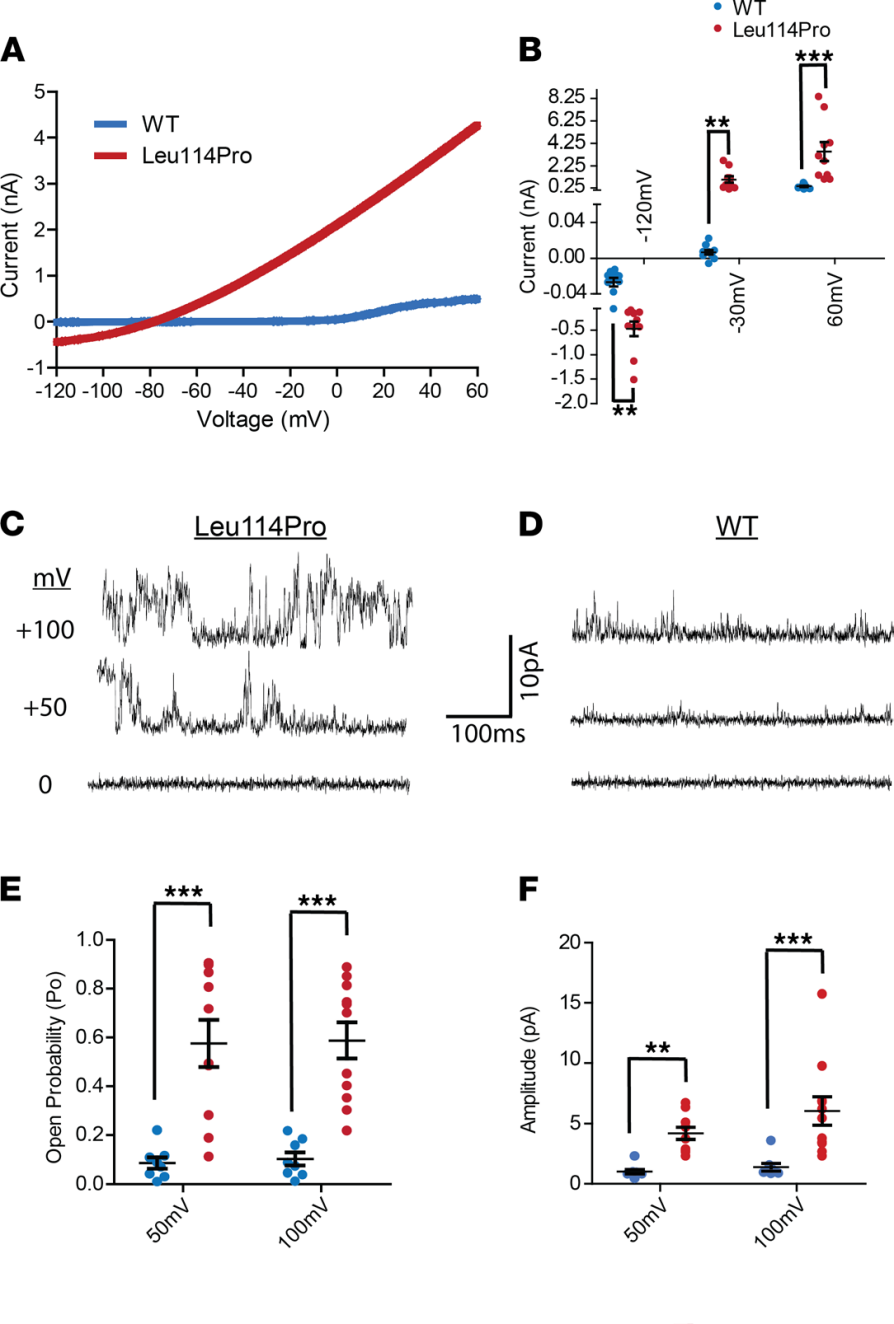
TALK-1 Leu114Pro causes a drastic gain of function in TALK-1 K^+^ current. (**A**) K^+^ currents monitored from TALK-1 WT or TALK-1 Leu114Pro with whole-cell voltage clamp recordings, (**B**) in response to a voltage ramp from –120 mV to 60 mV (mean ± SEM; *n* = 11 control cells; *n* = 10 TALK-1 Leu114Pro cells). (**C** and **D**) Single-channel plasma membrane K^+^ currents monitored through TALK-1 Leu114Pro or TALK-1 WT with attached patch voltage clamp recordings in response to the indicated voltage steps. (**E** and **F**) Single-channel recordings were analyzed for (**F**) current amplitude and (**E**) channel open probability (mean ± SEM; *n* = 8 TALK-1 WT cells; *n* = 11 TALK-1 Leu114Pro cells; *t* test, **P < 0.01, ***P < 0.001).

K^+^ currents recorded using HEK293 cells transfected with either TALK-1 WT or TALK-1 Leu114Pro demonstrated that TALK-1 Leu114Pro caused a large increase in whole-cell K^+^ currents compared with TALK-1 WT (current at −40 mV: TALK-1 Leu114Pro 774.16 ± 218.75 pA vs. TALK-1 WT 2.48 ± 1.86 pA) (Figure 2, A and B, and Supplemental Figure 1). Individual TALK-1 channel activity showed a 3.6-fold increase in current amplitude and a 2.9-fold increase in open probability at 100 mV for TALK-1 Leu114Pro compared with TALK-1 WT (Figure 2, C–F) (19). As the reversal potential for TALK-1 potassium currents is approximately –79 mV under physiological conditions, but the K^+^ concentration utilized for the cell-attached single-channel recordings moved the equilibrium potential for K^+^ (E_K_) to 0 mV, the holding potentials of the recordings with these conditions are equivalent to physiological voltages of –81 mV for 0 mV, –31 mV for +50 mV, and 19 mV for +100 mV. The reversal potential for TALK-1 Leu114Pro currents (approximately –79 mV) is close to E_K_ (approximately –81 mV) for the HEK293 cell recordings; therefore, these results are consistent with the TALK-1 Leu114Pro channels being K^+^ selective. As this TALK-1 mutation causes MODY in a dominant manner, we also measured the TALK-1 current under heterozygous conditions by creating a TALK-1-WT-P2A-TALK-1-Leu114Pro construct, which allows for equivalent expression of the TALK-1 WT and TALK-1 Leu114Pro subunits. Currents from HEK cells transduced with the TALK-1-WT-P2A-TALK-1-Leu114Pro construct displayed significant increases in TALK-1 currents (shown in Supplemental Figure 2) equivalent to those of cells expressing the TALK-1 Leu114Pro alone (Figure 2). Therefore, heterodimeric TALK-1 channels with a Leu114Pro and WT version are likely to show significant gain of function and are predicted to cause β cell *V_m_* hyperpolarization.

### TALK-1 Leu114Pro inhibits glucose-stimulated β cell depolarization.

**Figure 3:**
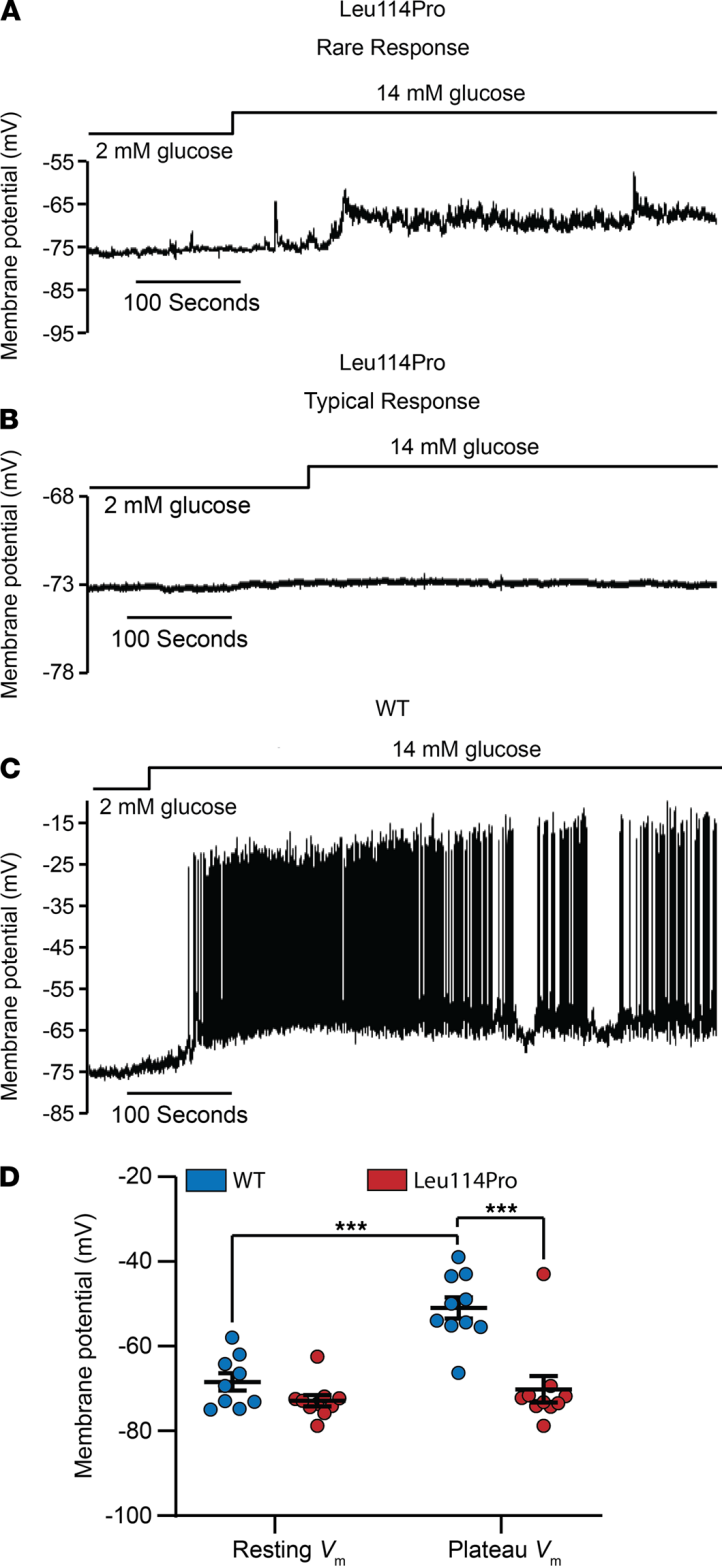
TALK-1 Leu114Pro hyperpolarizes the β cell *V_m_* and prevents action potential firing. (**A**–**C**) *V_m_* was monitored from β cells transduced with either TALK-1 WT or TALK-1 Leu114Pro in response to 2 mM or 14 mM glucose, as indicated in the figure. TALK-1 Leu114Pro displayed 2 types of responses: (**A**) one that occurred only in a rare subset of cells and (**B**) one that was more typical. (**D**) Average resting and plateau *V_m_* were calculated for TALK-1 Leu114Pro and TALK-1 WT (mean ± SEM; *n* = 10 control cells; *n* = 10 TALK-1 Leu114Pro cells; 1-way ANOVA followed by Bonferroni’s multiple comparison, ***P < 0.001).

Glucose-stimulated β cell electrical activity was monitored to determine how TALK-1 Leu114Pro affects *V_m_*. β Cells expressing TALK-1 WT showed glucose-stimulated *V_m_* depolarization to a plateau *V_m_* from where action potentials fired, typical of β cell electrical excitability. In contrast, a majority of β cells expressing TALK-1 Leu114Pro (80%, 8 of 10 cells) displayed neither *V_m_* depolarization nor action potential firing following glucose stimulation (Figure 3, A–D), which suggests that increased hyperpolarizing K^+^ conductance through channels containing a TALK-1 Leu114Pro subunit can hold β cells in a more hyperpolarized state. Interestingly, a small number of β cells expressing TALK-1 Leu114Pro (20%, 2 of 10 cells) showed modest glucose-stimulated *V_m_* depolarization; however, of these, only 1 cell fired action potentials (Figure 3B and Supplemental Figure 3). As β cells within an islet are electrically connected via gap junctions and TALK-1 Leu114Pro inhibits excitability in most β cells, this suggests that TALK-1 Leu114Pro would significantly blunt glucose-stimulated activation of VDCCs and Ca^2+^ influx.

### TALK-1 Leu114Pro reduces β cell Ca^2+^ influx and ER Ca^2+^ stores.

**Figure 4:**
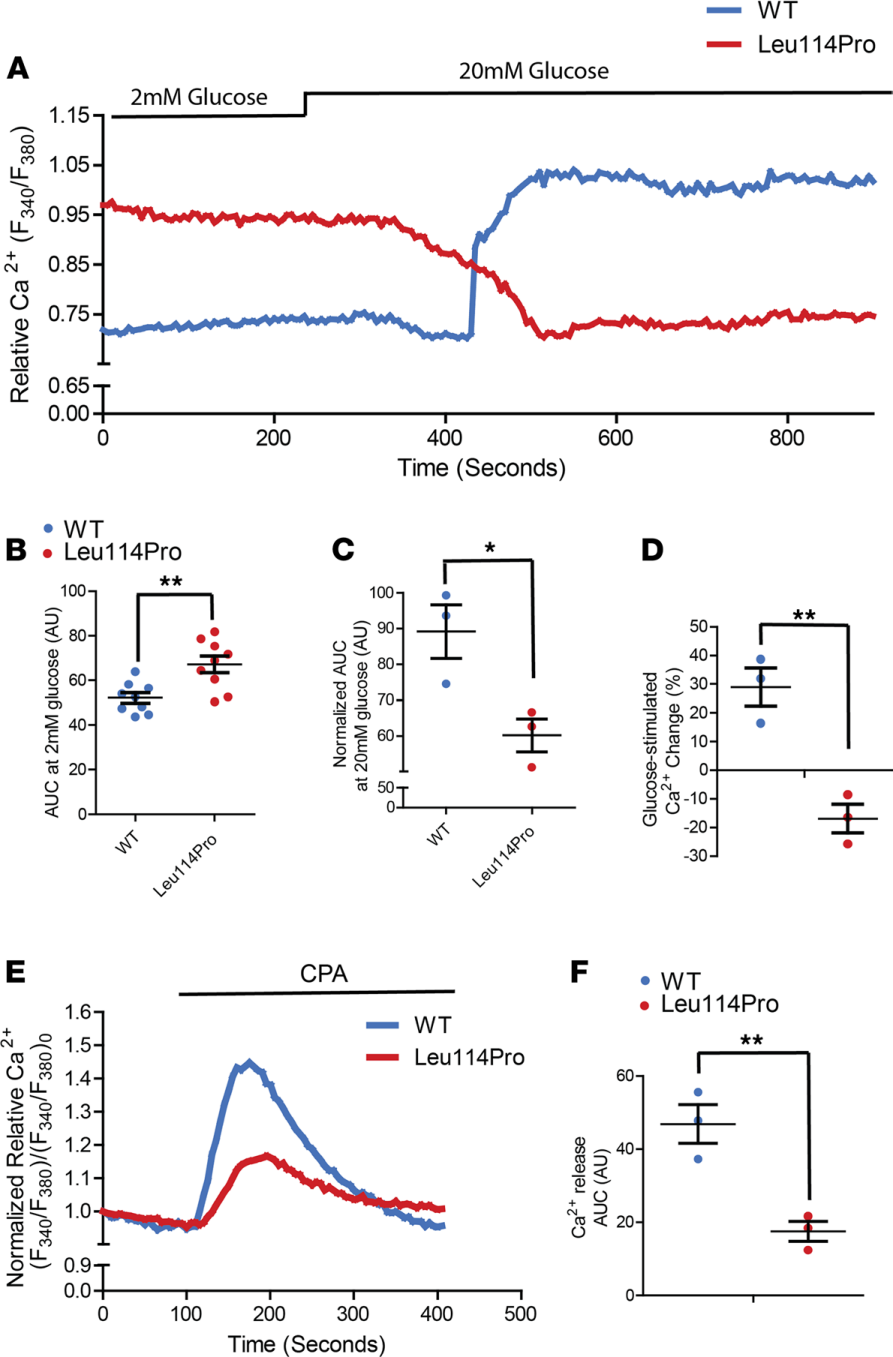
TALK-1 Leu114Pro modulates β cell Ca^2+^ homeostasis. (**A**) Representative β cell Ca^2+^ measurements in response to 2 mM and 20 mM glucose. (**B** and **C**) Area under the curve (AUC) analysis of β cell Ca^2+^ under (**B**) low (2 mM) glucose and (**C**) high (20 mM) glucose conditions. (**D**) AUC percentage change from low glucose to high glucose. (**E**) Representative β cell Ca^2+^ measurements in response to [Ca^2+^]_ER_ depletion by CPA and (**F**) the AUC analysis of the CPA response. Each dot corresponds to the average Ca^2+^ response for 1 animal. Mean ± SEM; (**C**, **D**, and **F**) *n* = 3 animals for TALK-1 WT and TALK-1 Leu114Pro Ca^2+^ experiments, (**B**) except for the 2 mM condition that included *n* = 9 animals (*t* test, *P < 0.05, **P < 0.01).

Ca^2+^ handling was monitored in mouse β cells following transduction of either TALK-1 WT or TALK-1 Leu114Pro. Glucose-stimulated (20 mM) β cell Ca^2+^ influx was abolished by expression of TALK-1 Leu114Pro (Figure 4, A–D). TALK-1 has been previously shown to modulate endoplasmic reticulum (ER) Ca^2+^ (Ca^2+^_ER_) homeostasis by providing a countercurrent for Ca^2+^_ER_ release (20); thus, TALK-1 Leu114Pro control of Ca^2+^_ER_ storage was also examined. Inhibition of SERCAs with cyclopiazonic acid (CPA) resulted in significantly less Ca^2+^_ER_ release in β cells expressing TALK-1 Leu114Pro compared with TALK-1 those expressing WT (62.6% decrease; Figure 4, E and F), suggesting reduced Ca^2+^_ER_ storage with TALK-1 Leu114Pro (20). β Cells expressing TALK-1 Leu114Pro also showed elevated basal cytoplasmic Ca^2+^ ([Ca^2+^]_cyto_) compared with β cells expressing TALK-1 WT (28.8% increase in AUC; Figure 4, A and B). Taken together, this suggests that, under basal conditions, TALK-1 Leu114Pro enhances Ca^2+^_ER_ leak, thereby increasing basal [Ca^2+^]_cyto_. Furthermore, the usual transient drop in β cell [Ca^2+^]_cyto_ following glucose stimulation of Ca^2+^_ER_ uptake (termed phase-0) (21) was amplified with TALK-1 Leu114Pro compared with TALK-1 WT (Figure 4, A and D). These changes would be predicted to reduce GSIS substantially. However, there was a small subset of β cells expressing TALK-1 Leu114Pro that did show glucose-stimulated [Ca^2+^]_cyto_ influx and would be predicted to have some GSIS (glucose-stimulated [Ca^2+^]_cyto_ influx was observed in 10 of a total of 99 islet cell clusters imaged from 3 animals, ≥22 islet clusters were imaged per animal; Figure 3B and Supplemental Figure 3).

### TALK-1 Leu114Pro reduces GSIS.

**Figure 5:**
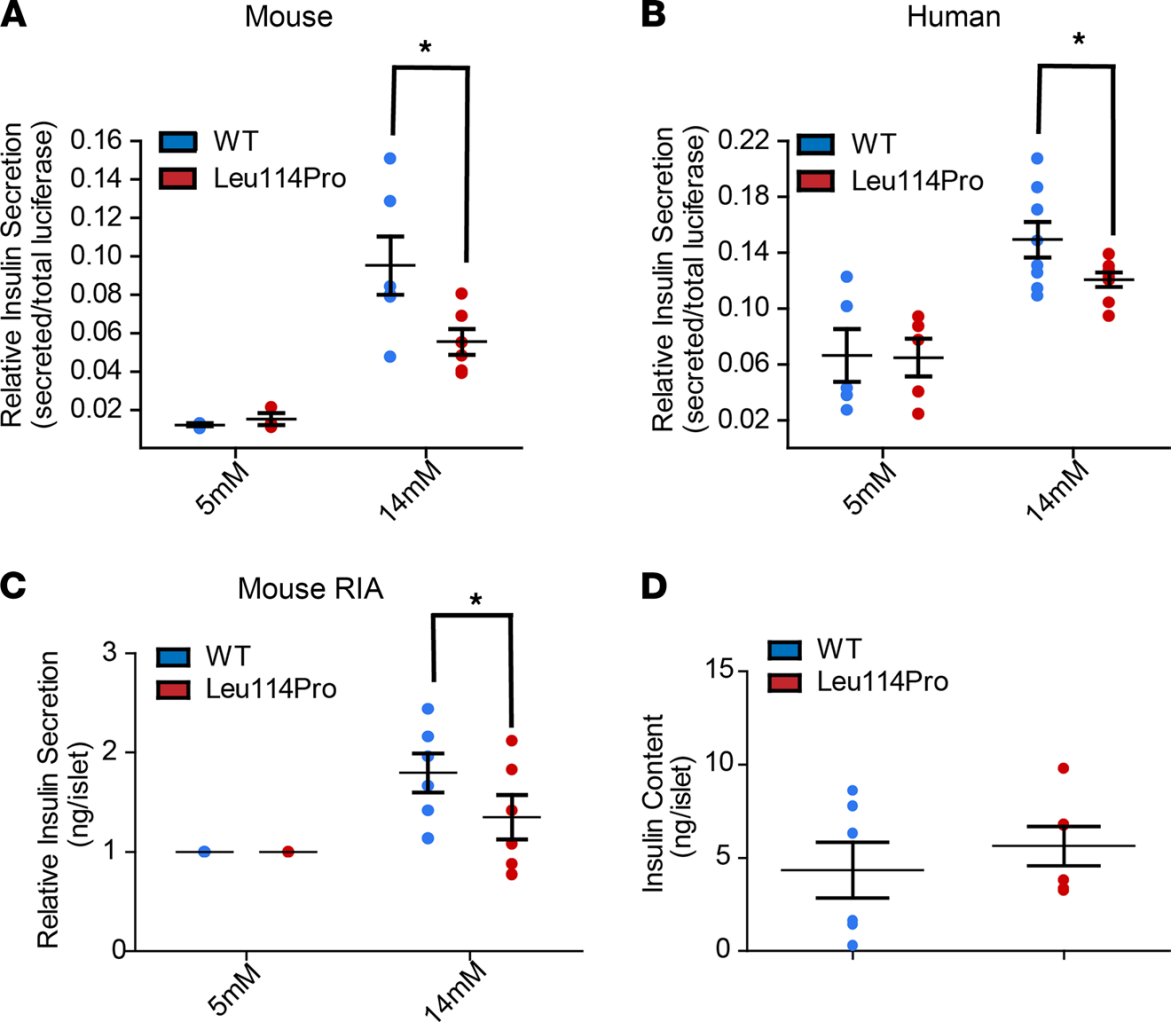
TALK-1 Leu114Pro reduces glucose-stimulated insulin secretion. (**A**) Mouse and (**B**) human islets transduced with viruses selectively expressing either TALK-1 WT or TALK-1 Leu114Pro and the NanoLuc-proinsulin luciferase insulin reporter. Islets were monitored for total secreted luciferase following exposure to 5 mM or 14 mM glucose. A radioimmunoassay was run on islets transduced with viruses selectively expressing either TALK-1 WT or TALK-1 Leu114Pro (**C**) to measure insulin secretion in response to 5 mM and 14 mM glucose as well as (**D**) total insulin content. Mean ± SEM; *n* = 6 animals (14 mM glucose, **A**), *n* = 3 animals (5 mM glucose, **A**); *n* = 8 human donors (14 mM glucose, **B**), *n* = 5 human donors (5 mM glucose, **B**), *n* = 6 animals (**C** and **D**) (*t* test, *P < 0.05).

To monitor insulin secretion specifically from β cells transduced with either TALK-1 WT or TALK-1 Leu114Pro, we first employed viral constructs containing a p2A proinsulin luciferase reporter where the insulin c-peptide has been replaced with luciferase (22). Using this assay, β cells expressing TALK-1 Leu114Pro showed comparable basal (5 mM glucose) insulin secretion but reduced GSIS (14 mM glucose) compared with those expressing TALK-1 WT, in both mouse (52% decrease in GSIS) and human (38% decrease in GSIS) islets (Figure 5, A and B). However, this proinsulin luciferase plasmid is not yet the standard for measuring insulin secretion; therefore, we also measured insulin secretion from mouse islets using a standard radioimmunoassay. Similar to the proinsulin luciferase assay, islets expressing TALK-1 Leu114Pro had reduced GSIS compared with TALK-1 WT islets (Figure 5C). These changes in insulin secretion were not due to changes in total β cell insulin content, which were equivalent in mouse islets expressing either TALK-1 Leu114Pro or TALK-1 WT (Figure 5D). However, as these were short-term experiments, prolonged inhibition of β cell insulin secretion in individuals with MODY carrying the TALK-1 Leu114Pro mutation may result in increased insulin content.

## Discussion

We have identified what we believe to be the first family with MODY due to a pathogenic variant in *KCNK16*. This TALK-1 gain-of-function p.Leu114Pro variant increases β cell K^+^ efflux, resulting in membrane hyperpolarization; alters β cell Ca^2+^ handling; and decreases GSIS. These data highlight the critical role of TALK-1 in β cell physiology. Unlike the only other MODY-associated K^+^ channelopathy (i.e., K_ATP_ channel dysfunction), TALK-1 is unresponsive to sulfonylureas (10). Thus, our data suggest a potentially novel therapeutic target for not only KCNK16-associated MODY but possibly for other forms of diabetes also.

TALK-1 belongs to the K2P channel family characterized by constitutive K^+^ flux, which serves critical roles in setting the *V_m_* of electrically excitable cells. The *KCNK16* transcript encoding TALK-1 is the most abundant K^+^ channel transcript in the human β cell (12, 13), and *KCNK16* shows the most islet-selective expression of all ion channel transcripts (11, 23). Similar to other K^+^ channels, such as K_ATP_ (24), TALK-1–mediated hyperpolarization of mouse and human β cell *V_m_* limits VDCC activity, Ca^2+^ entry, and GSIS (10). However, the K_ATP_ K^+^ conductance is significantly greater than the small constitutive conductance of TALK-1 (10, 11, 19, 24). Thus, TALK-1 mainly regulates the β cell *V_m_* following glucose stimulation when K_ATP_ channels close: their activity limits islet Ca^2+^ oscillation frequency and hence GSIS (10). A gain-of-function TALK-1 mutation would be predicted to affect glucose tolerance, adversely resulting in hyperglycemia, as demonstrated here.

Due to the almost complete suppression of glucose-stimulated depolarization and [Ca^2+^]_cyto_ influx in most β cells following expression of gain-of-function TALK-1 channels (Figures 3 and 4), β cells expressing the TALK-1 Leu114Pro channel were predicted to have a greater reduction of GSIS than what they displayed. However, 1 of 9 β cells expressing TALK-1 Leu114Pro showed glucose-stimulated action potential firing and 10% of β cells expressing TALK-1 Leu114Pro showed glucose-stimulated [Ca^2+^]_cyto_ influx; therefore, these β cells could be the source of the higher-than-expected GSIS (Figure 3B and Supplemental Figure 3). The mechanism for how a subset of β cells expressing TALK-1 Leu114Pro is able to undergo depolarization and [Ca^2+^]_cyto_ influx is unclear but may be due to β cell heterogeneity (25). Interestingly, the small number of β cells responding normally could explain the modest inhibition of GSIS by TALK-1 Leu114Pro and may also explain why individuals with TALK-1 Leu114Pro only require basal levels of insulin. Future studies using a mouse model with the TALK-1 Leu114Pro mutation and/or human β cells with TALK-1 Leu114Pro (potentially through CRISPR editing) may enable a clearer assessment of the impact of TALK-1 Leu114Pro on GSIS.

The *KCNK16*-containing locus is strongly associated with T2DM in multiple genome-wide association studies, including populations of differing ethnicities (17, 18, 26–28), with strongest association (*P* < 2 *×* 10^–8^) observed with the common nonsynonymous polymorphism rs1535500 (minor allele frequency [MAF] = 0.41, gnomAD database, subjects of non-Finnish European descent). The protein change (p.Ala277Glu) affects the C-terminal tail of TALK-1 and causes a modest (1.4-fold) increase in TALK-1 channel current, with both enhanced open probability and increased cell surface localization (10). The risk haplotype is also associated with increased expression of the adjacent gene *KCNK17*, which encodes another K2P channel, TALK-2 (29). TALK-2 is also expressed in islet cells with high specificity, though lower than TALK-1 (islet expression specificity index for *KCNK16* = 0.98 and for *KCNK17* = 0.76) (29). It is possible that the association of this locus with T2DM may be driven by more than one mechanism (29), i.e., that overactive TALK-1 and overexpression of TALK-2 may both contribute to hyperpolarization of the β cell *V_m_*, reducing glucose-stimulated Ca^2+^ influx and GSIS. We acknowledge that we have not assessed any potential regulatory role of the currently identified variant on expression of *KCNK17* (or of any other gene). However, that association is observed with a common variant and T2DM in the same gene (*KCNK16*) in which we have identified a rare variant associated with MODY indicates that the encoded protein (i.e., TALK-1) is not functionally redundant, and raises the possibility of TALK-1 as a therapeutic target for T2DM.

Mutations in K2P channels causing dramatic changes in K^+^ channel currents typically affect the pore domains of these channels (30–32). For example, loss-of-function mutations in the first or second pore domains of *KCNK3* (respectively, p.Gly97Arg and p.Gly203Asp) cause pulmonary hypertension (30). Similarly, a loss-of-function mutation in the first pore domain of TASK-2 (p.Thr108Pro) causes Balkan endemic nephropathy (31). A gain-of-function mutation (p.Gly88Arg) in the first pore domain of TALK-2, coded by *KCNK17*, causes a severe cardiac arrhythmia (32) and is the only previously identified disease-associated mutation in TALK channels.

Gain-of-function mutations in K_ATP_ significantly increase β cell K^+^ flux, resulting in neonatal diabetes and MODY (8, 9). In contrast, TALK-1 p.Leu114Pro results in a more modest diabetes phenotype, despite the 300-fold increase in whole-cell TALK-1 activity. This may be because TALK-1 activation shows voltage dependence (10, 11). Unlike K_ATP_, which is active at all voltages, TALK-1 is an outward rectifying channel that shows increased activation during depolarization (10, 11). Therefore, a gain of function in TALK-1 would be most active after β cell depolarization — limiting, but not abrogating, insulin secretion. The p.Leu114Pro mutation does increase TALK-1 current near the resting *V_m_* (Figure 2A and Supplemental Figures 1 and 2); however, this current is still less than the total β cell K_ATP_ conductance under euglycemic conditions. These data are in concordance with the proband’s clinical phenotype, with dramatic glucose elevation after an oral glucose load but only a modest increase in fasting plasma glucose.

TALK-1 is expressed on both the β cell plasma membrane and the ER membrane (20). Ca^2+^_ER_ release is balanced by negative charge on the luminal ER membrane; this charge is dissipated by ER TALK-1 K^+^ influx, leading to enhanced Ca^2+^_ER_ release (20). Thus, overactive TALK-1 channels (e.g., TALK-1 Ala277Glu) increase Ca^2+^_ER_ release, whereas TALK-1 ablation reduces Ca^2+^_ER_ release (20). Similarly, TALK-1 Leu114Pro likely increases basal [Ca^2+^]_cyto_ by promoting Ca^2+^_ER_ release; however, this modest elevation in basal [Ca^2+^]_cyto_ does not lead to increased basal insulin secretion. While this is presumably due to the small increase in basal β cell [Ca^2+^]_cyto_ caused by TALK-1 Leu114Pro, another possibility is that the location of Ca^2+^_ER_ release that is promoted by TALK-1 Leu114Pro activity may not be in the vicinity of insulin granules (33). Importantly, elevated β cell Ca^2+^_ER_ release under hyperglycemic conditions also results in ER stress, contributing to β cell dysfunction (34). TALK-1 Leu114Pro may contribute to β cell dysfunction via ER stress, as observed in some MODY subtypes (e.g., *INS* mutations in MODY-10; ref. 35); however, this remains speculative. Additionally, although highly β cell specific, TALK-1 is also expressed in human pancreatic δ cells, where it negatively regulates somatostatin release (36). TALK-1–KO mice show increased somatostatin secretion under low and high glucose conditions due to enhanced Ca^2+^_ER_ release (36); thus, a gain-of-function mutation in TALK-1 may reduce δ cell somatostatin secretion. The glycemic effects of this would be complex, given the inhibitory effect of somatostatin on both insulin and glucagon secretion (36), and require future investigation.

Some MODY subtypes (e.g., *ABCC8*, *KCNJ11*, *HNF1α*, and *HNF4α* MODY) are manageable through K_ATP_ inhibition (8, 37, 38) — i.e., sulfonylurea use. Although β cell *V_m_* depolarization with sulfonylureas may allow greater VDCC activity, potentially increasing insulin secretion in affected individuals in this family, TALK-1 itself is not sensitive to sulfonylureas (10). Further, and as detailed above, TALK-1 primarily modulates β cell *V_m_* during active insulin secretion when K_ATP_ is closed (i.e., during hyperglycemic conditions) (10). Thus, K_ATP_ inhibition may not completely normalize β cell *V_m_* or insulin secretion in individuals with TALK-1 gain-of-function MODY. This raises the possibility of TALK-1 inhibition as a druggable target. Genetic evidence, whether from rare (e.g., MODY) or common (e.g., T2DM, refs. 17, 18, 26–28) human disease, is a strong predictor of future successful drug development (39). Thus, our data have important therapeutic implications for potentially testing TALK-1–selective inhibitors for treatment of not only TALK-1 MODY, but also the far more common form of diabetes T2DM.

The proband in this family was able to transition successfully from low-dose insulin to metformin and dietary manipulation to achieve reasonable glycemic control (HbA1C 6.3%). However, when pregnant (a state of insulin resistance) she again required insulin. Her twin sister, mother, aunt, and grandmother (Figure 1A) all require insulin, from 9–25 units daily (detailed in Supplemental Results *Extended Clinical Data*). Variability in insulin doses within families with MODY been reported previously, particularly in MODY types associated with β cell ER stress (40).

In common with almost all monogenic diseases mapped using massively parallel sequencing technologies, we used exome sequencing as our mapping modality. In considering this choice, most Mendelian disorders arise from coding mutations (41), and most new genes mapped for Mendelian disorders using massively parallel sequencing technologies have been mapped through exome sequencing or through analysis of the exome within whole-genome sequencing data. Very few monogenic disorders have been identified as arising from noncoding or splice-site variants (42). All MODY genes identified to date are associated with mutations in the coding region or within the intron/exon boundary (±5 bp), regions which are captured well with exome sequencing (43). Although this does not necessitate that all MODY cases will arise from coding mutations, a priori exome sequencing is a strategically parsimonious and validated approach for new gene discovery. We acknowledge the inherent circularity (i.e., if only coding regions of the genome are assessed, then only coding variants will be identified) and the challenges of interpreting functionality of noncoding variants: both issues have potential to create bias in the literature. Nonetheless, *KCNK16* encodes a protein with a well-established role in β cell function and insulin secretion, and we have clearly demonstrated the pathogenic effects of our identified variant. However, we acknowledge that, to date, a further unrelated family with MODY with a pathogenic variant in *KCNK16* segregating with MODY phenotype has not been identified to our knowledge. We hope that the data presented here may lead to screening of *KCNK16* in families with MODY negative for mutations in other known MODY genes, as identification of a second family would further strengthen *KCNK16* as a MODY gene.

In conclusion, we have identified a mutation in *KCNK16* that causes a gain of function in TALK-1 and reduces glucose-stimulated Ca^2+^ influx, Ca^2+^_ER_ storage, and GSIS, resulting in MODY. TALK-1 is the first ion channel linked to MODY after K_ATP_, and it is expressed more selectively in islet cells compared with K_ATP_. The *KCNK16* locus is associated with T2DM risk in the general population. Our data suggest TALK-1 as an efficacious and islet-selective therapeutic target for both *KCNK16*-associated MODY and, potentially, T2DM.

## Methods

### Clinical recruitment.

A family with apparent autosomal dominant diabetes was recruited to a genetics study of MODY.

### Exome sequencing.

Exome sequencing, pipeline processing, quality control, and variant curation were performed as previously described (43) (detailed in Supplemental Methods). Exome data from the proband was analyzed for good-quality likely damaging rare variants in known MODY genes (1), using a conservative MAF threshold of < 0.001, based on (a) prevalence of pediatric diabetes of 0.2% (44) and (b) prevalence of MODY mutations in 2% of a pediatric diabetes population (3); further, most MODY mutations are private. Exome sequence data from the pedigree were analyzed for previously unreported and rare (MAF < 0.001) good-quality variants, of potentially damaging consequence, affecting highly conserved bases with appropriate segregation (i.e., heterozygous in affected individuals, absent in unaffected individuals).

### Plasmids and transient expression.

Human TALK-1 WT and TALK-1 Leu114Pro constructs were created by site-directed mutagenesis and then cloned into a vector containing a P2A cleavage site followed by mCherry (or TALK-1 Leu114Pro, for the TALK-1 WT P2A TALK-1 Leu114Pro construct) (Supplemental Figure 4). HEK293 cells, which have no endogenous TALK-1 expression, were transfected with 2 μg DNA using Lipofectamine 3000 (Life Technologies). Transfection efficacy was assessed and quantified using mCherry fluorescence (Supplemental Methods and Supplemental Figure 5).

### Lentivirus production.

HEK293 cells (Thermo Fisher, R70007) were transfected with lentivirus-producing plasmids; the plasmids used included the packaging plasmid (pCMV-dR7.74psPAX2), envelope plasmid (pMD2.G), and an expression plasmid (detailed in Supplemental Methods and Supplemental Figure 4). Lentivirus-containing supernatants were collected 3 days after transfection and used for transduction of primary β cells; after transduction, equal TALK-1 expression was confirmed by equivalent mCherry expression (detailed in Supplemental Methods and Supplemental Figure 6).

### Electrophysiological current recordings.

TALK-1 channel currents were recorded in HEK293 cells using a whole-cell voltage-clamp technique with an Axopatch 200B amplifier and pCLAMP10 software (Molecular Devices), as previously described (10). Briefly, voltage-clamp mode on an Axopatch 200B amplifier (Molecular Devices) was used to measure whole-cell TALK-1 currents. A Digidata 1440 was used to digitize currents that were low-pass–filtered at 1 kHz. Cells were washed with a Krebs-Ringer–HEPES buffer (KRHB) containing 119 mmol/L NaCl, 2 mmol/L CaCl_2_, 4.7 mmol/L KCl, 25 mmol/L HEPES, 1.2 mmol/L MgSO_4_, 1.2 mmol/L KH_2_PO_4_, and 11 mmol/L glucose, adjusted to pH 7.35 with NaOH. Patch electrodes (3–5 MΩ) were loaded with intracellular solution containing 140 mmol/L KCl, 1 mmol/L MgCl_2_, 10 mmol/L EGTA, 10 mmol/L HEPES, and 4 mmol/L Mg ATP (pH 7.25 with KOH). Whole-cell voltage-clamp recordings were done at room temperature (approximately 25°C). To record whole-cell TALK-1 currents in HEK cells, a lentiviral plasmid was produced containing a CMV promoter expressing TALK-1 WT followed by a P2A cleavage site and mCherry. Single-channel current recordings of TALK-1 were recorded with a cell-attached voltage-clamp technique also as previously described (19). Briefly, electrodes were pulled to a resistance of 8–10 megaohms and then coated with Sigmacote (Sigma-Aldrich). Extracellular solution contained 135 mM NaCl, 5 mM KCl, 1 mM MgCl_2_, 1 mM CaCl_2_, and 10 mM Hepes (pH 7.3 with NaOH). Intracellular pipette solution contained 150 mM KCl, 1 mM MgCl_2_, 5 mM EGTA, and 10 mM HEPES (pH 7.3 with KOH). Single-channel current openings were analyzed for open probability [P(o)] and current amplitude (pA) during a 5-second period of stimulation with 100 mV, 50 mV, and 0 mV using Clampfit software. Specifically, the event detection capabilities of Clampfit were used to identify single-channel openings in reference to the baseline of the trace. The currents of the identified single-channel openings were then averaged for pA, and the time spent in the open state versus total time was used to calculate P(o) (additional information on current recordings in Supplemental Methods and Supplemental Figures 1 and 2).

### β Cell V_m_ recordings.

Mouse islet cell clusters (10–20 cells) transduced with TALK-1 WT-P2A-mCherry or TALK-1 Leu114Pro-P2A-mCherry were washed twice with KRHB with 119.0 mmol/L NaCl, 2.0 mmol/L CaCl_2_, 4.7 mmol/L KCl, 25.0 mmol/L HEPES, 1.2 mmol/L MgSO_4_, and 1.2 mmol/L KH_2_PO_4_ (adjusted to pH 7.4 with NaOH) supplemented with either 2 or 14 mM glucose and cultured in KRHB for 20 minutes at 37°C, 5% CO_2_. Patch electrodes (4–6 MΩ) were filled with *V*_m_ IC with 140.0 mmol/L KCl, 1.0 mmol/L MgCl_2_, and 5.0 mmol/L HEPES (adjusted to pH 7.2 with KOH) supplemented with 20 μg/mL amphotericin B. The *V*_m_ of individual mCherry-positive β cells within islet cell clusters (10–20 cells) was recorded in current clamp mode using an Axopatch 200B amplifier with pCLAMP10 software. The electrical activity of patched β cells was recorded in response to treatments indicated in figure legends. Cells were identified as β cells if electrical activity ceased with 2 mM glucose.

### Islet and β cell isolation.

Islets were isolated from mouse pancreata from C57BL/6J (The Jackson Laboratory) and TALK-1–deficient C57BL/6J mice as previously described (10). Human islets from nondiabetic adult donors were provided by isolation centers of the Integrated Islet Distribution Program (donor information, Supplemental Table 1). Some islets were dispersed into cell clusters and then cultured for 12 to 18 hours (10). Cells were maintained in RPMI 1640 with 15% FBS, 100 IU/mL penicillin, and 100 mg/mL streptomycin in a humidified incubator at 37°C with an atmosphere of 95% air and 5% CO_2_.

### Calcium handling measurements.

Islets were incubated for 25 minutes in RPMI supplemented with Fura-2, AM (Molecular Probes), followed by incubation in KRHB with 2 mmol/L glucose for 20 minutes (10). For [Ca^2+^]_cyto_, Ca^2+^ imaging was performed as previously described (10), switching from 2 mM glucose to 20 mM glucose. For Ca^2+^_ER_, islets were perfused in KRHB buffer without extracellular Ca^2+^ and 100 μM diazoxide and monitored for Ca^2+^_ER_ release mediated through blockade of the sarco(endo)plasmic reticulum Ca^2+^-ATPase (SERCA) with 50 μM CPA (Alomone Labs), as previously described (20).

### Insulin secretion measurements.

Islets were transduced with lentiviruses containing a RIP promoter (45) upstream of either TALK-1 WT or TALK-1 Leu114Pro followed by a P2A cleavage site and NanoLuc-proinsulin (Supplemental Figure 4) (22, 46). Importantly, NanoLuc is cosecreted with insulin, enabling measurement of insulin secretion specifically from cells expressing either the TALK-1 WT or TALK-1 Leu114Pro construct (46). Briefly, islet clusters were cultured in a 96-well plate at 20 islet equivalents per well. The islet clusters were starved for 1 hour in 100 μL media (DMEM) containing 5 mM glucose before the assay was run. The assay was then run using the protocol for Promega Nano-Glo Luciferase Assay System. In short, the starvation media was replaced with fresh media containing 5 mM glucose and the islet clusters were allowed to secrete for 1 hour. The media were then collected and replaced with media containing 14 mM glucose, and the islet clusters were again allowed to secrete for 1 hour. After the secretion the cells were lysed and collected. The total lysate, the 5 mM glucose secretion media, and the 14 mM glucose secretion media were mixed with the Promega Nano-Glo substrate and imaged for luminescence using a BioTek Synergy H4 Hybrid Microplate Reader. Luminescence from the low and high glucose secretion media were divided by total luminescence from the lysate.

### Availability of sequencing data.

Availability of sequencing data generated and analyzed during this study is restricted to preserve patient confidentiality. However, on request the corresponding author will detail these restrictions and any conditions under which access to some data may be provided to bona fide researchers (subject to ethical approval).

### Statistics.

Functional data were analyzed using pCLAMP10 or Microsoft Excel and are presented as mean ± SEM. Statistical significance was determined using 2-tailed Student’s *t* test or a 1-way ANOVA followed by Bonferroni’s multiple comparison where appropriate. A 2-sided *P* value less than or equal to 0.05 was considered statistically significant.

### Study approval.

The study protocol was approved by the relevant human research ethics committee (Princess Alexandra Hospital, approval HREC/12/QPAH/109). All living family members gave written informed consent.

## Author contributions

ELD, MAB, SRJ, SMG, and DAJ contributed to project design. ELD, AMML, and SRJ acquired samples from family members. ELD, MAB, and SRJ contributed to wet work in the genetics lab, including conventional and massively parallel sequencing and DNA extraction. ELD, MAB, PJL, AMML, SRJ, and MM conducted bioinformatic analysis. SMG, DAJ, AYN, PKD, MTD, KEZ, and CMS were responsible for all of the cellular biology, including plasmid creation, lentivirus culture, voltage clamping, assessment of calcium flux, islet cell culture, and measurement of GSIS. SMG, DAJ, AYN, PKD, and MTD also conducted ion channel lab data analysis. SMG, SRJ, DAJ, and ELD were responsible for writing the manuscript and conducting critical review of the manuscript.

## Supplementary Material

Supplemental data

## Version 1 05/25/2021

In-Press Preview

## Version 2 07/08/2021

Electronic publication
