# Supplementary material for: A KCNK16 mutation causing TALK-1 gain of function is associated with maturity-onset diabetes of the young
Source: JCI Insight. 2021 Jul 8;6(13):e138057. doi: 10.1172/jci.insight.138057 (PMC8410089; doi:10.1172/jci.insight.138057)
Supplement: Supplemental data [file jciinsight-6-138057-s173.pdf]

Supplementary Appendix

Table of Contents	Page Number
Supplementary Methods	2
Supplementary Results	
• Clinical Data	6
• Table S1	7
• Table S2	8
• Table S3	9
• Figure S1	11
• Figure S2	12
• Figure S3	13
• Figure S4	14
• Figure S5	15
• Figure S6	16
Supplementary References	17

Supplementary Methods

Exome sequencing and analysis

DNA was extracted from blood or saliva and sequencing libraries constructed according to each manufacturer's instructions. Exome capture was performed using either Nimblegen SeqCap EZ Human v3.0 Exome Enrichment Kit for the proband (Roche, Basel, Switzerland) or Nextera^{TM} Rapid Capture Exome kit for subsequent family members (Illumina, San Diego, CA, USA). Pre- and post-capture quality and yield were assessed by Agilent High Sensitivity DNA assay (Agilent, Santa Clare, CA) using Bioanalyzer 2100 (Agilent, Santa Clare, CA) and by qPCR using the KAPA Library Quantification Kit (Kapa Biosystems, Wilmington, MA).

Multiplexed massively parallel sequencing (six samples per flowcell lane) was performed on an Illumina HiSeq 2000 for the proband or HiSeq 2500 for additional family members (Illumina, San Diego, CA, USA), generating 100-base pair paired-end reads.

Data were demultiplexed using Illumina Data Analysis Pipeline software (Bcl2fastq v1.8.4) and aligned to the current reference human genome (hg19, released February 2009) using the Novoalign alignment tool (V3.00.02). Sequence alignment files were converted using Picard tools (v1.124). Variants were called using the Genome Analysis Toolkit (GATK v2.7-2) and annotated using ANNOVAR. Further analysis of sequence data was performed using custom scripts employing R and Bioconductor.

After quality control and exclusion of artefact, good quality variants with minor allele frequency (MAF) <0.05 (assessed against internal and external databases, including ExAC(1), 1000 Genomes(2), and dbSNP137(3)) were retained for analysis.

Lentiviral Plasmids

Lentiviral plasmids expressing human TALK-1 WT and TALK-1 Leu114Pro were designed for HEK cell and β-cell studies; these plasmids were created by site-directed mutagenesis and then cloned into a vector containing a P2A cleavage site followed by mCherry or TALK-1 Leu114Pro. A cartoon representation of the different lentiviral constructs that were utilized in these experiments is presented in **Supplementary Figure S1**. For whole-cell current recordings, a lentiviral plasmid was created that contains the CMV enhancer and CMV promoter placed immediately upstream of either TALK-1 WT or TALK-1 Leu114Pro followed by a P2A cleavage site and mCherry. For the β-cell Ca^{2+} experiments, a lentiviral plasmid was

created that contains the β-cell specific RIP1-mini-CMV promoter[4] placed immediately upstream of either TALK-1 WT or TALK-1 Leu114Pro. To utilize the Nano-Luc Pro-insulin Luciferase(4) as a read-out of insulin secretion we developed lentiviral plasmids that contained the same RIP1-mini-CMV Promoter mentioned above expressing either TALK-1 WT or TALK-1 Leu114Pro followed by a P2A cleavage site and the Nano-Luc Pro-insulin Luciferase. A CMV promoter expressing GFP was also inserted into the plasmid to enable visualisation of plasmid expression. The sequences for TALK-1 are those corresponding to transcript variant 3 (NM_001135105). Transcript Variant 3 was used for these experiments because it is the most highly expressed ion channel producing variant of *KCNK16* in human β-cells.

Lentivirus Production

HEK293FT (Thermo Fisher, R70007) cells were cultured in DMEM GlutaMax with 10% FBS, 100IU/mL penicillin, and 100mg/mL streptomycin to 50-70% confluency in 100mm dishes. The media was then switched to DMEM GlutaMax with 10% Heat-Inactivated FBS for transfection. Chloroquine was added to the cells and allowed to sit for 10 minutes. The following DNA mixture (46.7ul of 2.5M $CaCl_2$, 18.65ug lentiviral plasmid, 13.9ug packaging plasmid (pCMV-dR7.74psPAX2), and 5.56ug envelope plasmid (pMD2.G) brought up to 470ul with water) was added to 470ul of 2X HBS and then added dropwise onto the cells. Cells were incubated with DNA mixture for 5-7 hours and then the transfection media was aspirated off and replaced with fresh DMEM GlutaMax with 10% FBS, 100IU/mL penicillin, and 100mg/mL streptomycin. Media containing lentivirus was harvested 72 hours later and frozen at −80°C before use in mouse and human islet cells.

Lentiviral Transduction

For viral transduction, islets were isolated from mouse pancreata and dispersed into clusters through titration in 0.005% trypsin. After overnight recovery, the islet clusters were transduced using polybrene with 3rd-generation lentiviruses containing the above-mentioned lentiviral plasmids. The virus was left on the cells for 5-7 hours and then replaced with fresh media. The virus was then allowed to express for 2-3 days. The mCherry fluorescence allowed us to identify β-cells that were successfully transduced and only mCherry positive cells were selected for analysis. On average, the mCherry fluorescence, and therefore expression level, was similar between TALK-1 WT and TALK-1 Leu114Pro expressing cells **(Supplementary Figure S3)**.

TALK-1 Whole-Cell Currents

Voltage-clamp mode on an Axopatch 200B amplifier (Molecular Devices) was used to measure whole-cell TALK-1 currents. A Digidata 1440 was used to digitize currents that were low-pass–filtered at 1 kHz. To record whole cell TALK-1 currents in HEK cells, a lentiviral plasmid was produced containing a CMV promoter expressing TALK-1 WT followed by a P2A cleavage site and mCherry. HEK cells were transfected with 2ug of the lentiviral plasmid DNA using Lipofectamine 3000. The mCherry allowed us to identify cells that were successfully transfected and currents were recorded from cells with similar levels of mCherry fluorescence. On average, the mCherry fluorescence, and therefore expression level, was similar between TALK-1 WT and TALK-1 Leu114Pro expressing cells **(Supplementary Figure S2)**. The whole cell currents were analysed using ClampFit and Excel.

TALK-1 Single Channel Currents

Single Channel currents were measured in HEK cells using attached-patch voltage-clamp technique. Electrodes were pulled to a resistance of 8 to 10 megaohms and then coated with Sigmacote. Extracellular solution contained 135mM NaCl, 5mM KCl, 1mM $MgCl_2$, 1mM $CaCl_2$, and 10mM Hepes (pH 7.3 with NaOH). Intracellular pipette solution contained 150mM KCl, 1mM $MgCl_2$, 5mM EGTA, and 10mM HEPES (pH 7.3 with KOH). Single channel current openings were analysed for open probability and current amplitude during a 5-second period of stimulation with 100mV, 50mV, and 0mV. A threshold of 0.75pA was set to determine channel openings. Our reported amplitude and open probability for TALK-1 WT is similar to that previously published for TALK-1(5, 6).

Calcium Imaging

Islets were incubated at 37°C for 25min in RPMI supplemented with 2µmol/L Fura-2, AM (Molecular Probes), followed by incubation in Krebs-Ringer–HEPES buffer with 2mmol/L glucose for 20min. Cells were then imaged every 5 second using a Nikon Eclipse Ti2 microscope equipped with a Photometrics Prime 95B 25mm sCMOS Camera. The ratios of Fura-2AM fluorescence excited at 340 and 380 nm (F340/F380) were measured. The cells were perifused at a flow rate of 2 mL/min at 37°C with solutions containing glucose concentrations specified in the figures.

Nano-Luc Pro-insulin Luciferase Assay

Islet clusters were cultured in a 96-well plate at 20 islet equivalents per well. Islet clusters were transduced as described above with lentiviral plasmids that contained the RIP1-mini-CMV Promoter expressing either TALK-1 WT or TALK-1 Leu114Pro followed by a P2A cleavage site and the Nano-Luc Pro-insulin Luciferase. The islet clusters were starved for one hour in 100ul media (DMEM) containing 5mM glucose before running the assay. The assay was then run using the protocol for Promega Nano-Glo® Luciferase Assay System. In short, the starvation media is replaced with fresh media containing 5mM glucose and the islet clusters are allowed to secrete for one hour. The media is then collected and replaced with media containing 14mM glucose and the islet clusters are again allowed to secrete for one hour. After the secretion the cells are lysed and collected. The total lysate, the 5mM glucose secretion media, and 14mM glucose secretion media are mixed with the Promega Nano-Glo® substrate and imaged for luminescence using a BioTek Synergy™ H4 Hybrid Microplate Reader. Luminescence from the low and high glucose secretion media were divided by total luminescence from the lysate.

Human Islet Cells

Human islets were obtained for the Nano-Luc Pro-insulin Luciferase studies from the Integrated Islet Distribution Program (IIDP). Human donors were both male and female as well as from multiple ethnic backgrounds. Information on each human donor is presented in Supplementary Table S1.

Supplementary Results

Extended Clinical Data

The non-obese proband (individual IV.1) was diagnosed with antibody-negative diabetes age 15 years. Sanger sequencing screening for mutations *GCK, HNF1A* and *HNF4A* was negative. She has not had ketosis or other diabetes-related complications. She initially only required 1-2 units of basal insulin to maintain an HbA1c 5.7-6.5%. In more recent times this young woman (now aged 36) has been managed with diet and metformin; currently her HbA1c is 6.3% with elevated fasting glucose (6-7mmol/L). During pregnancy she was prescribed low dose (<5 units) insulin; despite persistent fasting hyperglycaemia (6-7.5) she did not tolerate high doses because of hypoglycaemia.

The proband's identical twin sister (individual IV.2) was diagnosed with diabetes contemporaneously with the proband. In contrast with her sister, this individual is managed with multiple daily injections, requiring 20-25 units of insulin daily. She has never experienced ketosis or diabetes-related complications. Other clinical information for this individual is unavailable.

The proband's mother (individual III.2) and aunt (individual III.2), both of normal weight, presented aged 15 and 13 years respectively with antibody-negative diabetes. Neither has experienced ketosis or diabetes-related complications after many decades. The proband's mother requires 8-11 units isophane insulin daily, maintaining an HbA1c of 6-7%. Her aunt requires 9-10 units of mixed insulin (Mixtard® 30/70 mane), maintaining an HbA1c <6%.

The proband's maternal grandmother (individual II.1) was diagnosed with diabetes during her first pregnancy (noting that this is often the first point of biochemical testing for otherwise well individuals), aged 30 years. She required <10 units/day of basal insulin, maintaining an HbA1c 6-6.5%. She recently died aged 82 years, without experiencing ketosis or diabetes-related complications.

The proband's maternal great-grandmother (individual I.1) also had diabetes. Further details of this individual are not available.

The proband's brother (individual IV.3), currently aged 34 years, does not have diabetes and is currently euglycaemic on finger-prick testing (no recent formal OGTT but previous formal OGTT which was negative).

Supplementary Tables and Figures

Table S1. Human islet donor information. Human islets received for human insulin secretion studies from the Integrated Islet Distribution Program.

Islet Isolation Center	Age	Gender	Ethnicity	Weight (kg)	Height (cm)	BMI	Cause of Death	Purity (%)	Average blood glucose in mmol/L
Southern California	53	Male	White	83.9	182.9	25.1	Stroke	96	6.2
Southern California	51	Male	White	76	177.8	24.0	Head Trauma	96	7.8
Miami	57	Female	Black	79.5	165.0	29.2	Stroke	95	9.6
Scharp/Lacy	50	Male	Hispanic	83.9	160.0	32.8	Anoxia	90	7.5
Scharp/Lacy	57	Male	Hispanic	72.6	167.6	25.8	Stroke	95	7.8
Wisconsin	53	Female	White	38.1	145.0	40.0	Stroke	92	7.0
Scharp/Lacy	45	Male	Black	106.1	180.3	32.6	Anoxia	95	7.3
Southern California	33	Male	White	106.4	179.0	33.2	Anoxia	80	7.8

*1 mg/dL = 0.0555 mmol/L

Table S2 Coverage statistics for exome sequencing

	MODY_II.1	MODY_III.1	MODY_IV.1	MODY_IV.3
**Total bases**	5835073129	5386986408	5187196181	1824158333
**% bases on target exons**	38.4	35.9	47.9	35.8
**Target exon median base coverage**	52.9	44.2	32.0	12.8
**Target exon mean base coverage**	64.4	53.5	37.6	14.2
**% target exon bases with coverage >1x**	98.2	98.6	91.0	95.5
**% target exon bases with coverage >5x**	93.6	94.5	94.4	90.4
**% target exon bases with coverage >10x**	88.0	88.7	79.2	80.1
**% target exon bases with coverage >30x**	65.9	63.0	54.6	59.9

Table S3: Bioinformatic filtering of identified variants

Filtering steps	MODY-II.1*	MODY-III.1*	MODY-IV.1*	MODY-IV.3
Variants identified with MAF≤0.05	7114	7389	8368	7320
Remaining variants of potentially damaging consequence** of good quality after removal of platform-related artefact	659	639	434	(NA)
Heterozygous variants remaining with MAF <0.001	343	333	213	(NA)
Remaining variants segregating appropriately with observed autosomal dominant inheritance	13			
Remaining variants with high conservation (GERP >2.5)	6			
Remaining variants predicted to be damaging[#]; MAF reported from ExAC	*KCNK16 (*NM_001135105) c.341T>C; p.Leu114Pro; MAF = 0<br>*USP42* (NM_032172) c.C3569G: p.Pro1190Arg; MAF=0<br>*KIAA1407* (NM_020817) c.2687G>A; p.Arg89Gln; MAF 0.00007 (rs144192553)			
Remaining variants in candidate genes known to be involved in insulin secretion	*KCNK16 (*NM_001135105) c.341T>C; p.Leu114Pro			

*affected individuals

**potentially damaging consequences defined as nonsynonymous single nucleotide variants (SNVs), splice site SNVs, frameshift substitution, stopgain or stoploss SNVs.

#Using protein prediction algorithms SIFT(7), Mutation Taster Human Splicing Finder v3.0(8) and PolyPhen2(9)

MAF: minor allele frequency. ExAC: Exome Aggregation Consortium. QC: quality control. GERP: genomic evolutionary rate profiling score.

Figure S1: Average whole cell TALK-1 current recordings in HEK cells expressing TALK-1 Leu114Pro vs. TALK-1 WT.

Average whole cell potassium currents from TALK-1 Leu114Pro-expressing cells (in red) is dramatically greater compared to TALK-1 WT expressing cells (in blue) **(Panel A)**. A zoomed-in graph of the average TALK-1 WT whole-cell current, which is similar to that of previously published TALK-1 WT currents(10) **(Panel B)**.

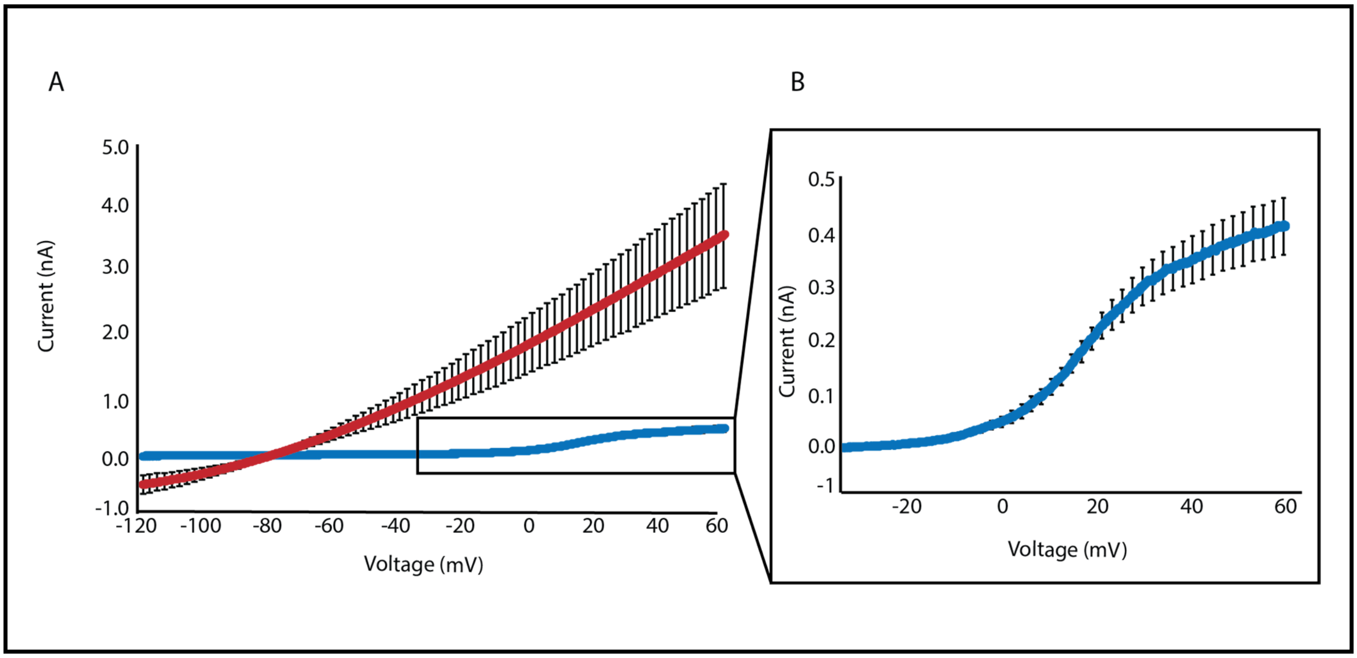

Figure S2: Average whole cell TALK-1 current recordings in HEK cells expressing TALK-1 WT P2A Leu114Pro construct vs. TALK-1 WT.

Average whole cell currents from TALK-1 WT P2A TALK-1 Leu114Pro expressing cells (in red) is dramatically greater than the K2P currents from cells expressing TALK-1 WT (in blue) **(Panel A)**. The mean K2P current recorded at -120 mV, -30 mV, and 60 mV for cells expressing either TALK-1 WT (blue bars, N= 11) or TALK-1 WT P2A TALK-1 Leu114Pro (red bars, N= 15) ± SEM **(Panel B)**.

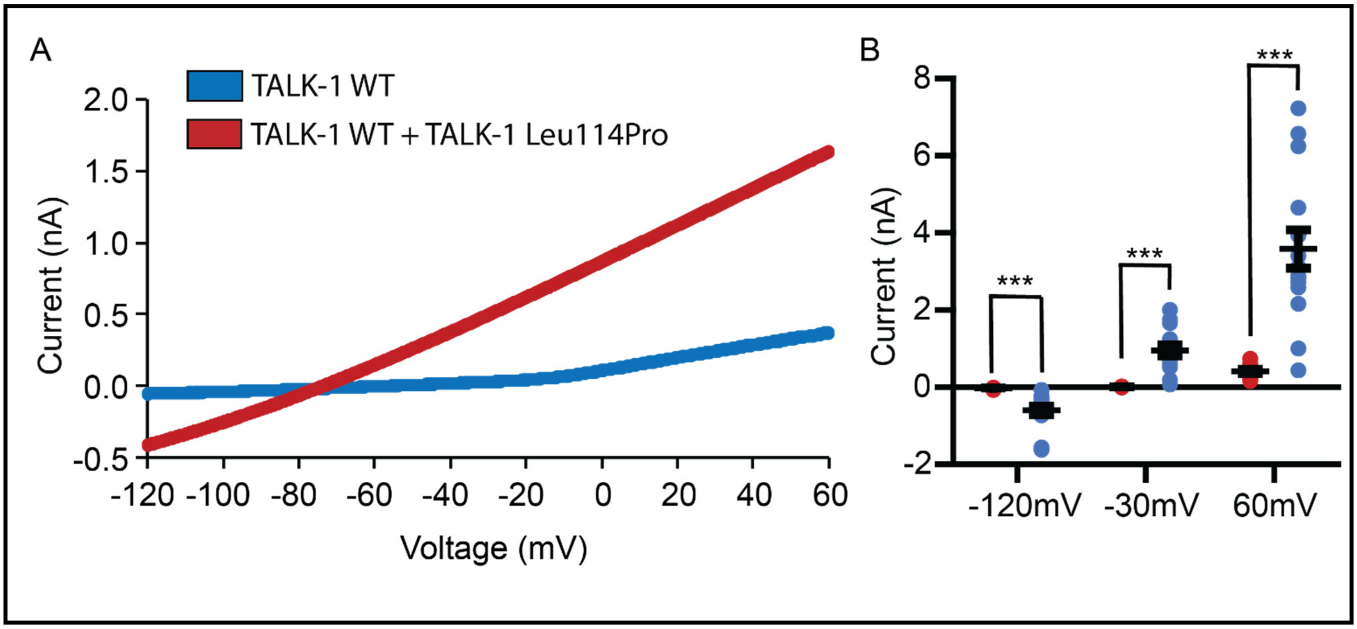

Figure S3: Representative traces of 10% of the TALK-1 Leu114Pro expressing β-cells that show glucose-stimulated action potential firing and $[Ca^{2+}]_{cyto}$ influx. Representative V_m recording from one of the TALK-1 Leu114Pro expressing β-cells that showed both V_m depolarization and action potential firing upon stimulation with 14mM glucose. The TALK-1 Leu114Pro construct used for these recordings contained a P2A mCherry; ROI intensity from this cell was equivalent to the other 90% of β-cells that did not respond to glucose with action potential firing **(Panel A)**. Representative $[Ca^{2+}]_{cyto}$ recording from one of the TALK-1 Leu114Pro expressing islet cell clusters that showed glucose-stimulated $[Ca^{2+}]_{cyto}$ influx **(Panel B)**. From a total of 99 islet cell clusters expressing TALK-1 Leu114Pro that were assessed for $[Ca^{2+}]_{cyto}$ influx from three mice, 10 responded to glucose with $[Ca^{2+}]_{cyto}$ influx (~10%). ROI intensity of mCherry fluorescence was also comparable between cells that responded to glucose with $[Ca^{2+}]_{cyto}$ influx and those cells expressing TALK-1 Leu114Pro that did not respond to glucose

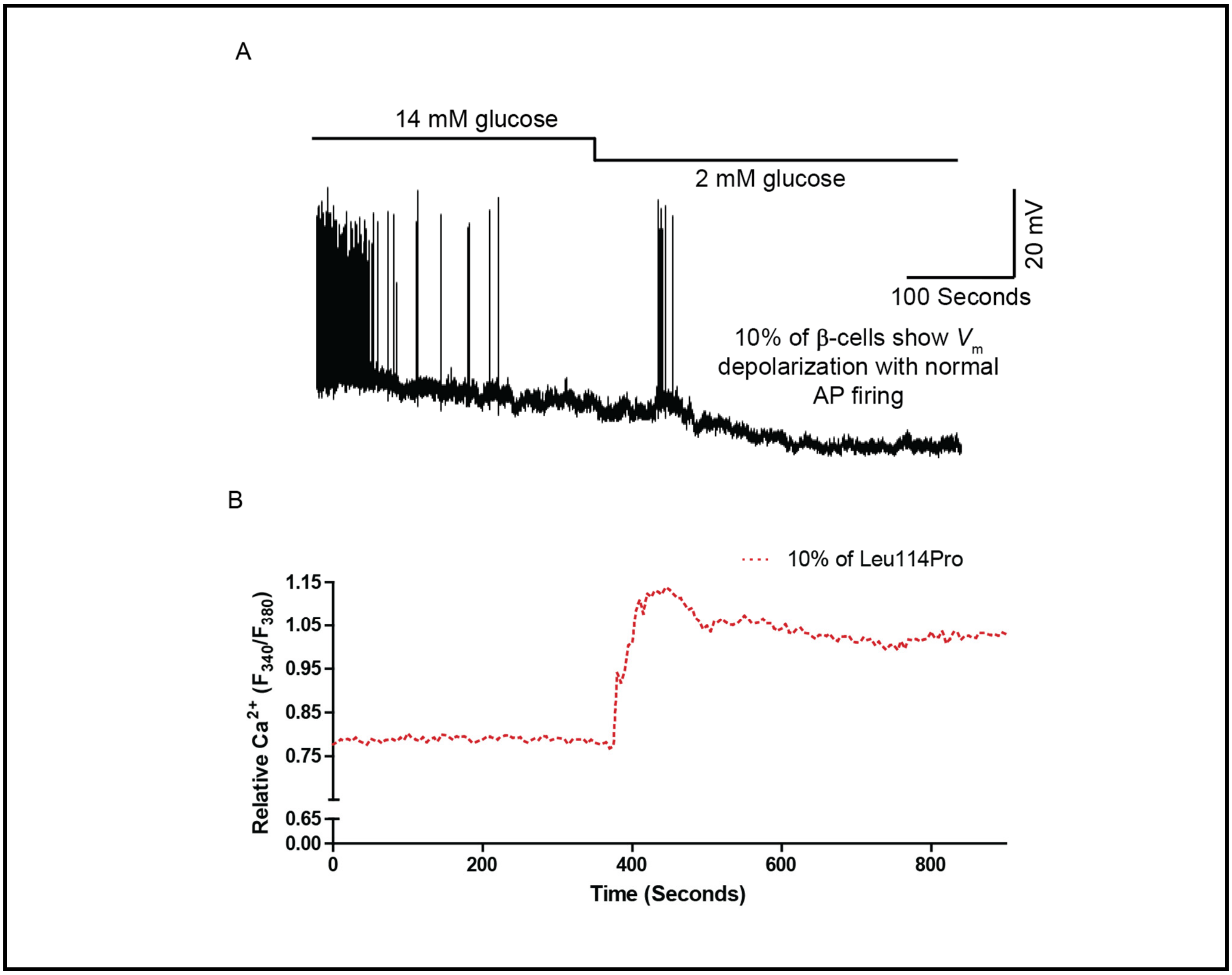

Figure S4: Lentiviral plasmids designed for TALK-1 WT, TALK-1 Leu114Pro, and TALK-1 WT P2A TALK-1 Leu114Pro **expression in HEK cells and β-cells.**

Lentiviral Plasmids were designed with either a CMV promoter or a Rat-Insulin Promoter expressing TALK-1 followed by a P2A cleavage site and mCherry (for electrophysiology and calcium experiments), pro-insulin luciferase (for insulin secretion experiments), or P2A cleavage site and TALK-1 Leu114Pro (for electrophysiology experiments in cells expressing equivalent amounts of TALK-1 WT or TALK-1 Leu114Pro). The TALK-1 cloned into these constructs were either Wild-Type (WT) or WT P2A TALK-1 Leu114Pro **(Panel A)** or TALK-1 Leu114Pro **(Panel B).** Plasmids were sequenced and chromatograms show the correct TALK-1 WT **(Panel C)** and TALK-1 Leu114Pro **(Panel D)** sequences.

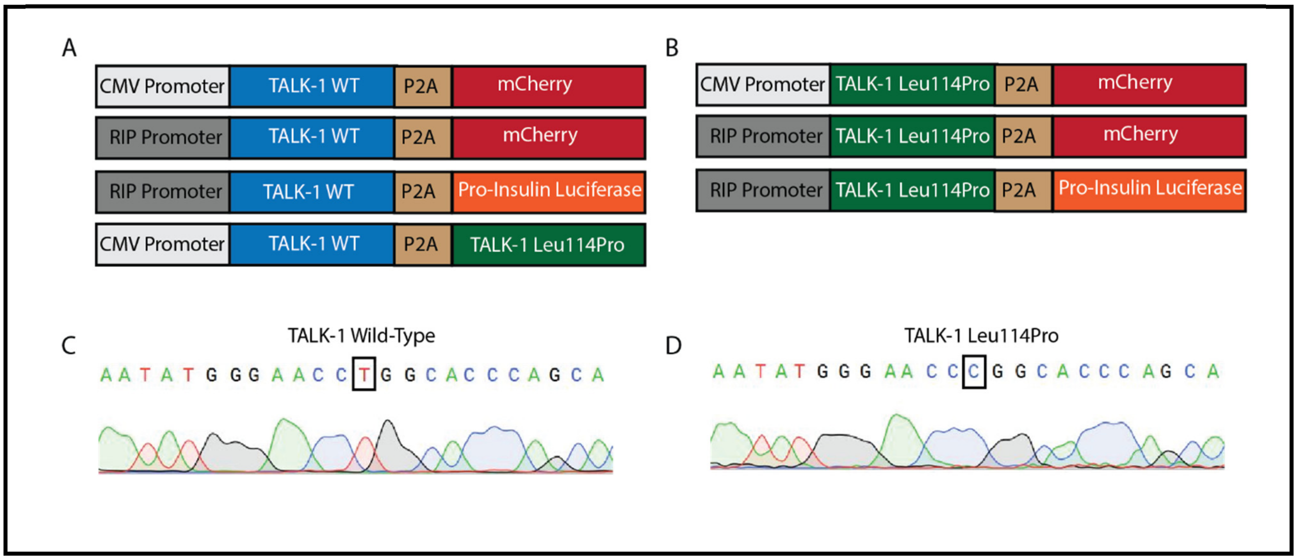

Figure S5: Expression of TALK-1 WT and TALK-1 Leu114Pro in HEK cells for current recordings.

HEK cells were transfected with constructs containing a CMV Promoter expressing TALK-1 followed by a P2A cleavage site and mCherry. TALK-1 expressing cells are represented by mCherry fluorescence (red) and all nuclei were stained with Hoescht (blue); cells expressing TALK-1 WT **(Panel A)** or TALK-1 Leu114Pro **(Panel B)** (images shown were taken at 20X magnification). Equivalent mCherry fluorescence in TALK-1 WT- and TALK-1 Leu114Pro-expressing cells indicates similar TALK-1 expression levels **(Panel C)** (N=30 for cells expressing TALK-1 WT; N=26 for cells expressing TALK-1 Leu114Pro).

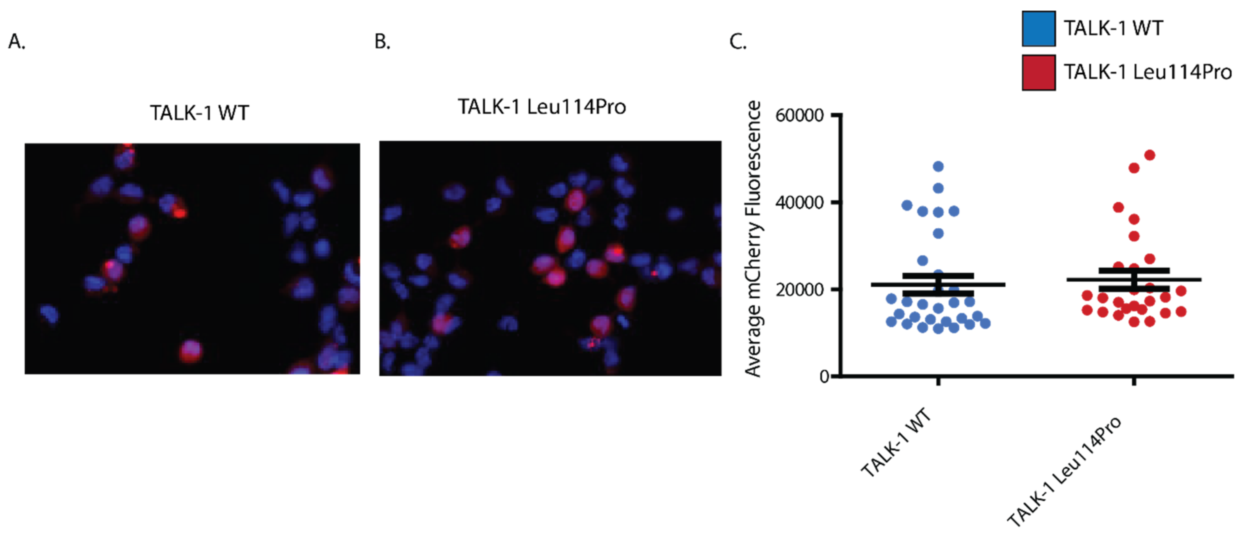

Figure S6: Expression of TALK-1 WT and TALK-1 Leu114Pro in mouse β-cells.

Dispersed islet cells were transduced with the viruses containing a Rat-Insulin Promoter expressing TALK-1 followed by a P2A cleavage site and mCherry. TALK-1 expressing β-cells (shown in red) imaged with a 10X objective compared to total islet cells (marked in blue) for TALK-1 WT **(Panel A)** and TALK-1 Leu114Pro **(Panel B)**. Average mCherry fluorescence shows similar expression levels between TALK-1 WT and TALK-1 Leu114Pro **(Panel C)** (N=48 for cells expressing TALK-1 WT; N=42 for cells expressing TALK-1 Leu114Pro).

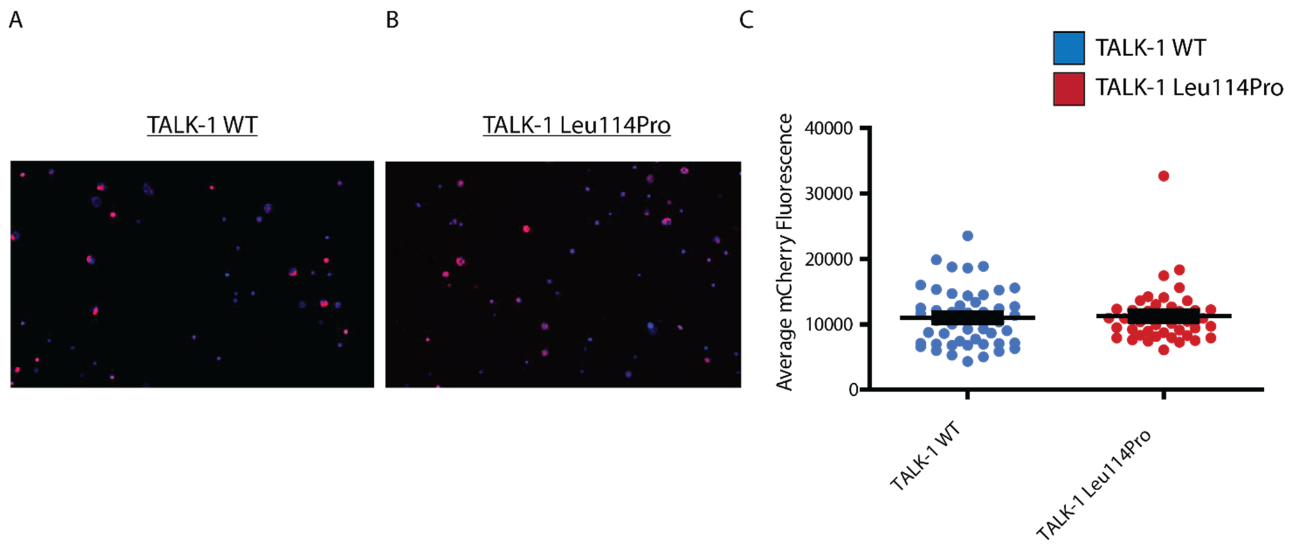

Supplementary References

1. Lek M, Karczewski KJ, Minikel EV, Samocha KE, Banks E, Fennell T, et al. Analysis of protein-coding genetic variation in 60,706 humans. *Nature.* 2016;536(7616):285-91.
2. Abecasis GR, Auton A, Brooks LD, DePristo MA, Durbin RM, Handsaker RE, et al. An integrated map of genetic variation from 1,092 human genomes. *Nature.* 2012;491(7422):56-65.
3. Sherry ST, Ward M, and Sirotkin K. dbSNP-database for single nucleotide polymorphisms and other classes of minor genetic variation. *Genome Res.* 1999;9(8):677-9.
4. Wang H, Bender A, Wang P, Karakose E, Inabnet WB, Libutti SK, et al. Insights into beta cell regeneration for diabetes via integration of molecular landscapes in human insulinomas. *Nature communications.* 2017;8(1):767.
5. Kang D, and Kim D. Single-channel properties and pH sensitivity of two-pore domain K+ channels of the TALK family. *Biochem Biophys Res Commun.* 2004;315(4):836-44.
6. Vierra NC, Dadi PK, Milian SC, Dickerson MT, Jordan KL, Gilon P, et al. TALK-1 channels control beta cell endoplasmic reticulum Ca2+ homeostasis. *Science signaling.* 2017;10(497).
7. Ng PC, and Henikoff S. Predicting deleterious amino acid substitutions. *Genome Res.* 2001;11(5):863-74.
8. Schwarz JM, Cooper DN, Schuelke M, and Seelow D. MutationTaster2: mutation prediction for the deep-sequencing age. *Nature methods.* 2014;11(4):361-2.
9. Adzhubei IA, Schmidt S, Peshkin L, Ramensky VE, Gerasimova A, Bork P, et al. A method and server for predicting damaging missense mutations. *Nature methods.* 2010;7(4):248-9.
10. Vierra NC, Dadi PK, Jeong I, Dickerson M, Powell DR, and Jacobson DA. Type 2 Diabetes-Associated K+ Channel TALK-1 Modulates beta-Cell Electrical Excitability, Second-Phase Insulin Secretion, and Glucose Homeostasis. *Diabetes.* 2015;64(11):3818-28.
